# Public sector engagement of private healthcare providers during the COVID-19 pandemic in Uttar Pradesh, India

**DOI:** 10.1371/journal.pgph.0000750

**Published:** 2022-07-22

**Authors:** Ankita Meghani, Shreya Hariyani, Priyanka Das, Sara Bennett

**Affiliations:** 1 Department of International Health, Johns Hopkins Bloomberg School of Public Health, Baltimore, Maryland, United States of America; 2 Johns Hopkins India Private Limited (JHIPL), Lucknow, Uttar Pradesh, India; Universidad Austral de Chile, CHILE

## Abstract

The COVID-19 pandemic has strained public health resources and overwhelmed health systems capacity of countries worldwide. In India, the private sector is a significant source of healthcare particularly in large states like Uttar Pradesh (UP). This study sought to examine: (i) the government’s approach to engaging the private health sector in the COVID-19 response in UP; (ii) the effects of government’s engagement on private providers’ practices and (iii) the barriers and facilitators to effective private sector engagement during the period. While the literature acknowledges weaknesses in private sector engagement during emergencies, our study provides deep empirical insight into how this occurs, drawing on the UP experience. We reviewed 102 Government of UP (GOUP) policy documents and conducted 36 in-depth interviews with government officials, technical partners, and private providers at district- and state-levels. We developed timelines for policy change based on the policy review and analyzed interview transcripts thematically using a framework analysis. We found that GOUP’s engagement of the private sector and private providers’ experiences varied substantially. While the government rapidly engaged and mobilized private laboratories, and enlisted private hospitals to provide COVID-19 services, it undertook only limited engagement of solo private providers who largely acted as referral units for suspected cases and reported data to support contact tracing efforts. Informal private providers played no formal role in the COVID-19 response, but in one district supported community-level contact tracing. Allopathic, alternative medicine, and diagnostic private providers faced common barriers and facilitators affecting their engagement relating to provider fear, communication, testing capacity, case reporting, and regulations. The establishment of mixed diagnostic networks during COVID-19 demonstrates the potential for public-private collaboration, however, our study also reveals missed opportunities to engage smaller-scale private health providers and establish mechanisms to effectively communicate and mobilize them during the pandemic, and beyond.

## Introduction

The coronavirus (COVID-19) pandemic has overwhelmed health systems capacity of countries worldwide as providers figure out how to treat an influx of COVID-19 cases while also managing care for other patients. The COVID-19 pandemic has strained health care resources creating challenges for diagnostics and treatment, as well as surveillance, and quarantine. India, which is home to 1.3 billion people [1], confronts a unique challenge with regard to managing the pandemic: controlling infections is difficult given high population density in urban slums where about 35% of the urban population resides [2], but so too is providing services for the nearly two out three Indians who live in rural areas [3]. While compared to many high income countries India’s COVID-19 caseload and number of deaths to-date appears relatively low, there is likely under-reporting [4].

India’s public health system aims to provide health services at little to no cost, but the government spends only 0.96% of its GDP on health, which is one of the lowest in the world [5]. Inadequate health care funding has routinely left public health facilities understaffed, with about 70% of health professionals working in the private sector and 75% of all health professionals located in urban areas [6]. Underfunding has also resulted in limited access to health equipment, such as ventilators in public health facilities [7]. A recent comparison between private and public sector health capacity showed that the private sector has greater intensive care unit capacity in terms of number of beds, and ventilators compared to the public sector [8].

India’s private sector is large, fragmented, and diverse. Informal providers, or experience-based practitioners, such as rural medical practitioners, are often the primary source of care among the rural and urban poor [9]. They are a diverse group varying in training, skillset, and types of health conditions they treat [9]. Formal providers include two main types: (i) allopathic providers who have been formally trained in medicine with at least a Bachelor of Medicine, Bachelor of Surgery (MBBS); and (ii) alternative medicine practitioners, who are trained in Ayurveda, Yoga and Naturopathy, Unani, Siddha and Homeopathy (AYUSH) and have degrees in Bachelor of Ayurveda Medical and Surgery (BAMS) and Homeopathic Medicine and Surgery (BHMS) and Unani Medicine and Surgery (BUMS).

Not surprisingly, the limited access to public sector healthcare has contributed to both higher levels of care-seeking and out-of-pocket expenditure in the private sector [10, 11]. About 55% of all Indians seek care in the private sector [12]. There is also substantial evidence to show the high reliance of low-income households on private health care providers: in India as a whole about 40% of hospitalizations among the poor are in private health care facilities, and in Uttar Pradesh (UP), the most populous state in India, this rises to about 51.4% [11]. Given the private sector’s important role in healthcare delivery, understanding its engagement in the COVID-19 response is critical.

At the national and state levels, the Government of India (GoI) leveraged existing policy initiatives to engage the private sector as a partner in the COVID-19 pandemic. For example, the Ayushman Bharat health insurance scheme, which provides the poorest with free healthcare in both public and private health facilities, made additional provisions for its insurees to access private sector COVID-19 testing and treatment at no cost [13]. Similarly, the government engaged larger private hospitals to provide COVID-19 treatment. While government strategies seemed to focus on engaging larger private hospitals, its approach to engaging smaller scale providers that the poor use was less clear.

Emerging findings from an analysis of low-and middle-income countries in Sub-Saharan Africa, South Asia and the Middle East early in the pandemic describe the strain placed on the private health sector due to declining numbers of patients. Smaller scale private providers, such as doctors running their own practice, and small nursing homes and hospitals were particularly hard hit [14]. Financial pressures have resulted from loss of income due to low patient load, the inability to provide a full range of services as well as investments in the procurement of personal protective equipment (PPEs) [14]. Recent World Health Organization (WHO) guidance also emphasizes the issues in engaging the private sector during the COVID-19, and documenting existing experiences and emerging evidence to inform future private sector engagements [15]. To-date however, reports on how the private health sector has been engaged in humanitarian or emergency response point to sporadic, reactive and uncoordinated engagement [16, 17], rather than a more systematic approach.

The goal of our study is to provide actionable evidence, that can guide how government collaborates with and leverages private sector health care capacity to meet healthcare needs during COVID-19 within the specific context of UP, India. In this study, we sought to understand: (i) the government’s approach to engaging a range of private sector health providers particularly those located within poorer and rural communities in the COVID-19 response; (ii) the effects of government’s engagement on private providers’ practices and (iii) the barriers and facilitators to effective private sector engagement. A deeper understanding of the private sector’s experiences and needs during the pandemic can help inform the development of effective approaches to engage the private sector.

## Methods

We conducted a case study in the state of UP, with two embedded district level cases, for which we purposively selected two eastern UP districts: Prayagraj and Gorakhpur. Our study focused on access to health services among low income and rural populations, including migrant laborers, so our district selection was based on: (i) high number of COVID-19 cases; (ii) large migrant population size who were returning to their villages following the national wide lockdown; and (iii) large rural population in the district. At the time of data collection, Prayagraj and Gorakhpur respectively had the third and fourth highest COVID-19 disease burden in the state and were experiencing a high influx of returning migrant laborers. Additionally, both districts are very populous and rural (see **Table 1**).

**Table 1:** Characteristics of embedded district cases.

Characteristics	Gorakhpur	Prayagraj
Cumulative COVID-19 disease burden^a^	4^th^ highest out of 75 UP districts (2,898)	3^rd^ highest out of 75 UP districts (3,726)
Migrant population^b^	14^th^ highest out of 75 UP districts (20,913)	2^nd^ highest out of 75 UP districts (90,943)
% rural population^c^	80%	75% Largely rural with a large urban center
Population size^c^	10^th^ highest out of 75 UP districts 4,440,895	Highest out of 75 UP districts 5,954,391

^a^COVID-19 burden as of September 2020 [18]

^b^UNICEF Migrants Tracking Phase 2 Update (May 28 2020) [19]

^c^2011 Census of India population data for Gorakhpur [20] and Prayagraj [21]

Our study draws on two data sources: policy documents and in-depth interviews at the district- and state-levels.

### Policy document review

We conducted a policy document review to: (i) understand the government’s engagement of private health care providers, particularly smaller scale private healthcare providers in the COVID-19 response in UP; (ii) identify the range of approaches the government used to engage the private sector, and the nature of these engagements.

Policy documents were identified from a compendium of COVID-19 guidelines and government orders published and maintained by the Government of Uttar Pradesh (GOUP) [22]. We screened all the government guidelines published starting January 25, 2020 through till December 31, 2020. We selected for full data extraction those which (i) described guidelines or orders that specifically targeted the private health sector or (ii) described guidelines or orders that were applicable to the private health sector, even if not solely targeted at the private sector. Policy documents were excluded if they described information that exclusively targeted public sector health workers or were related to non-health sectors.

In sum, information from 102 GOUP orders and circulars were extracted into a structured spreadsheet, which focused on: (i) date of the policy order (ii) the content of the policy order (e.g., guidance on COVID-19 referral protocols, treatment, charges in the private sector hospitals, etc.); (iii) the agency/organization/institution responsible for implementing the order; and (iv) type of private sector stakeholder referred to in the policy (e.g., private clinics, AYUSH doctors, nursing homes, multispecialty hospitals).

### In-depth interviews

To better understand the policy context, we interviewed state-level respondents who were directly involved in organizing the state-wide COVID-19 response, including technical partners, as well as officials at the Department of Medical Health and Family Welfare, and the Department of AYUSH. These interviews were focused on eliciting the respondent’s perspective on steps taken by the government to engage the private sector and the challenges, if any, faced in this process. Also, where necessary, we used the interviews to seek additional insight about the content and rationale of the government orders. We conducted further interviews with district health officials in our two case study districts, who were directly involved in monitoring and implementing COVID-19 GOUP orders and circulars at the district-level. In these interviews we sought to learn about implementation processes for private sector engagement.

We also conducted interviews with private health providers in the two study districts, through which we sought to understand: (i) the government’s approach to engaging a range of different private sector health providers; (ii) the effects of government’s engagement on providers’ practices, e.g., their decisions to remain open or closed, and the types of health services they provided; and (iii) the barriers and facilitators to effective private sector engagement.

In each district, we sought to interview four types of private health provider respondents: (i) allopathic providers, who have received a MBBS degrees; (ii) AYUSH providers, who have received degrees in BAMS, BHMS, or BUMs; (iii) laboratory service providers, who are providers of COVID-19 tests and (iv) experience-based practitioners, often referred to as rural medical practitioners (RMPs), who have not received formal training.

Multiple strategies were used to identify potential respondents. We identified allopathic providers based on their affiliation with private health facilities reporting to the UP’s Health Management Information Systems. AYUSH providers were identified through online health directories. We also drew on our existing professional networks in Uttar Pradesh and on snowball sampling to be introduced to respondents across all four categories.

Overall, we conducted 36 in-depth semi-structured interviews with government officials, technical partners, and private providers at district- and state-levels (**Table 2**). Interviews employed a standard interview guide which had been adapted to different types of respondents (policy-maker/provider, and by type of provider) and were conducted in Hindi and English depending on the respondent’s preference. Prior to each interview, we sought written informed consent. Once consent was granted, the interview was conducted by phone or WhatsApp from October 2020 –January 2021, ranging from 25 minutes to 1.5 hours. The interviewer wrote up brief memos following each interview, and full recordings were transcribed, and translated into English.

**Table 2:** Study respondents at the district and state level.

Category of respondent	State-level	District-level
Government	3	3
Technical partners	2	-
Allopathic providers	-	13^b^
AYUSH providers	1^a^	8
Rural medical practitioners	2	1
Laboratory services	1	2^c^
**Total**	**9**	**27**

^a^Respondent was a representative of the AYUSH association

^b^Four respondents were also health association representatives at the local or state level

^c^One respondent managed laboratory services, and another was a pathologist running a private laboratory

In terms of analysis, we developed timelines for policy change based upon the policy document review. We analyzed the transcripts from the interviews thematically using a framework analysis [23]. After the study team had reviewed both the transcripts and the memos we discussed emerging themes and for the purposes of this paper decided to employ a simple analytical framework that aligned with our interview guide and study objectives, and captured four main categories: (i) the nature of government’s engagement with the private sector; (ii) the effects of government policies and approaches on private health providers’ practices during the pandemic; (iii) barriers to the effective engagement of the private sector; and (iv) facilitators of effective engagement of the private sector. We applied this analytical framework to all interview data for private providers and state level respondents. We then summarized our findings in separate memos written by type of private health care provider (allopathic; AYUSH; laboratory; RMP) incorporating material from state level respondents where relevant. This approach allowed us to compare findings across respondent groups.

### Ethical considerations

Johns Hopkins Bloomberg School of Public Health deemed this research as not human subjects research. The study was approved for ethical research by the Institutional Review Board of SIGMA Research and Consulting in New Delhi, India (10016/IRB/20-21).

## Results

### Overview of GOUP response to COVID-19 and the role of the private sector

The GOUP released more than 250 government orders between January-December 2020, including those shared by the national Ministry of Health and Family Welfare (MoHFW). A majority of these were issued between March and September 2020. Of these, 102 were identified as being relevant to the private sector. **Table 3** provides a timeline for the main government orders showing the private sector’s engagement in the COVID-19 pandemic. **S1 Table** provides a detailed analysis.

**Table 3:** Timeline showing key policy measures highlighting the role of the private sector during COVID-19.

Date	Description of government order/guideline
24 March 2020	Nation-wide lockdown resulted in suspension of all outpatient services in private facilities, however allowed pharmacies to remain open and private hospitals to provide non-COVID emergency services
28 March 2020	Pradhan Mantri Gareeb Kalyan Life Insurance package made available to all public and private health workers requisitioned to participate in the COVID-19 response
11 April 2020	Continuity of essential non-COVID health services (e.g., obstetric care and trauma) in the private hospitals and nursing homes
19 April 2020	Trainings on Infection Prevention and Control guidelines, biomedical waste, and other disinfection protocols to be ensured by district health authorities across all facilities (including private).
6 April 2020 9 April 2020 10 April 2020	All hospitals, including private designated as L1 and L2 facilities
19 April 2020	Network of private laboratories for COVID-19 testing expanded in accordance with the Indian Council of Medical Research and other quality assurance guidelines.
10 April 2020	Recommendation to enlist AYUSH Master trainers and paramedical staff to support district-level authorities
15 April 2020	COVID-19 testing at private labs capped at Rs. 4,500 per test
5 May 2020 20 May 2020	District level lists indicate designation of private and public hospitals as L1 and L2 COVID facilities^a^
14 May 2020	Guidelines outlining the purchase of PPE to the private sector
31 May 2020	National lockdown ended
16 June 2020	All outpatient services resumed in public and private hospitals; private clinics directed to adhere to outline infection prevention and control guidelines
18 June 2020	Cost of single-step RT-PCR tests for COVID-19 capped at Rs. 2,500 in Private Sector
19 June 2020	Treatment costs at designated COVID-19 private medical colleges and institutions under Ayushman Bharat; cost capped at Rs. 4,500 per day
21 July 2020	Home isolation allowed for COVID-19 positive cases
7 July 2020	COVID treatment costs fixed in private facilities fixed per national recommendations
24 July 2020	Mobile medical vans set up to collect samples from severe acute respiratory infection patients in private hospitals
10 September 2020	Cost of COVID tests in private sector capped at Rs. 1,600

^a^L1 and L2 facilities respectively managed non-critical and more critical COVID-19 patients

#### Timeline for key policy measures

In response to the exponential rise of COVID-19 cases in March, the GoI implemented a nationwide lockdown beginning on March 25, 2020 [24]. Policy orders immediately suspended private providers (of all types) from providing outpatient care, with exceptions made only for non-COVID emergency cases at private nursing homes and hospitals. Overnight, public health facilities became the primary source of care in the nation and were expected to provide COVID-19 and non-COVID-19 related health services as well as serve as quarantine facilities.

Beginning February 2020, the GOUP began sharing national- and state-level guidelines on a containment plan for COVID-19 along with health facility safety protocols and infection control measures for COVID-19. National and state-level Crisis Management Committees were established. In addition, state and district-level Rapid Response Teams were deployed to conduct active and passive surveillance across public and private facilities and establish containment zones, as needed.

At the end of March 2020, the Pradhan Mantri Gareeb Kalyan Package Insurance scheme for health workers fighting COVID-19 was launched. This provided life insurance coverage to all health workers in the public sector involved in the COVID-19 response, and those in the private sector who were requisitioned to participate in the response. Between April and June 2020, a large number of orders were issued addressing screening and testing strategies; data reporting processes; dedicated facilities for COVID-19 cases and suspected cases; training and deployment for the health workforce; and assessing capacity across public and private facilities in terms of number of personnel, available beds and testing facilities. The government also began cataloging the numbers of ventilators, intensive care unit beds available across the public and private sector.

Several of these orders explicitly sought to integrate private providers in the response (**Table 3**). For example, accredited private laboratories meeting the Indian Council of Medical Research’s (ICMR) guidelines were identified as testing sites, and some private hospitals were requisitioned as COVID-19 treatment facilities. The GOUP developed a three-tier system (Levels 1, 2, and 3) to manage COVID-19 facilities based on disease severity (non-critical patients to complex and critical COVID-19 cases). Private medical colleges were designated as L3 facilities and were also requisitioned to establish a COVID-19 testing facility. The treatment of all patients in these L3 facilities and other empaneled institutions was covered under the Ayushman Bharat scheme. Furthermore, facilities in private medical colleges and other private facilities empaneled under the Ayushman Bharat scheme, providing COVID-19 services, and received PPEs kits and masks at no cost. For other private facilities, the government outlined guidelines for purchasing masks and PPEs and provided a list of approved vendors.

GOUP issued guidance on infection control in health care facilities, targeting both public and private sectors. District-level committees were established to monitor proper implementation of the Infection Prevention and Control guidelines, and to ensure all health workers in public and private health facilities were trained. Districts provided trainings on these guidelines, which were required for private hospitals providing emergency services.

Realizing the adverse implications of the lockdown for non-COVID services, in early April the GOUP reiterated its order from March, encouraging private hospitals and nursing homes to reopen and provide essential health services for patients who had tested negative for COVID-19. Additional government guidelines in May underscored strengthening disease surveillance through better tracking of COVID-19 testing in the private sector and improving the readiness of COVID-19 health facilities.

Initially, all COVID-19 patients were quarantined in designated COVID-19 facilities. This implied that private practitioners who encountered patients suspected of suffering from COVID-19 had to refer them to government facilities for testing, quarantine, and care. This practice to document and notify district authorities of suspected COVID-19 cases continued even after private outpatient services resumed in June 2020, and after home quarantine was permitted in early July 2020. The GOUP released a number of guidelines for private health facilities on the follow-up and discharge of patients in home isolation. July 2020 also marked a pivotal point as the GOUP capped the charges of COVID-19 treatment in private hospitals, allowing private hospitals to directly admit, charge and treat COVID-19 patients.

Over the course of the pandemic, the GOUP issued a number of orders related to price regulation in the private sector to make testing more affordable and accessible to the public. For example, the price for COVID-19 testing was initially capped at Rs 4,500, and the price cap was lowered twice, reaching Rs 1,600 in early September. To expand access to COVID-19 testing, the GOUP also directed districts to set up sample collection booths in a number of public health facilities and establish mobile medical vans to collect samples from patients with severe acute respiratory infection in private hospitals.

### Engagement strategies by type of private provider

The GOUP’s approach to engaging the private sector during the COVID-19 pandemic varied substantially by provider type. Unlike more formally qualified providers–private laboratories, allopathic and AYUSH providers–the GOUP appeared to have made no effort to engage with RMPs. Furthermore, none of the government orders issued explicitly addressed this group. State-level key informants explained this lack of engagement was based upon the perceived capacity of such providers. For example, one respondent suggested that it would be inappropriate to *“pressurize people to do things which they cannot do*, *or they don’t want to do”* further noting that given the very sparse facilities such providers had, engaging them in the response *“might be spreading more than actually helping”* (State-level key informant 4).

In contrast, the GOUP rapidly recognized the need to engage private sector laboratories in scaling up access to affordable COVID-19 diagnostics early in the pandemic. Through a series of government orders in April 2020, the GOUP established the principles of engaging private laboratories (**Table 4**) placing diagnostics at the center of the COVID-19 response in UP. Further, to onboard new laboratories and diagnostics, the GOUP conducted trainings and provided free access to the External Quality Assurance Service program. At the district-level, respondents also appeared to be aware of these government orders and programs, and in one study district, a respondent even described a local diagnostics company being persuaded by the district health authorities to get accredited to build the district’s COVID-19 diagnostic capacity.

**Table 4:** Summary of relevant government orders from March-September 2020 describing the engagement of the private laboratories in the COVID-19 response in Uttar Pradesh, India.

Date	Relevant content from government order/guideline
16 March 2020	Established team to monitor private laboratories during the pandemic
4 April 2020	Described the role that private laboratory testing should play in areas where government laboratories do not exist. Stipulates that private labs should have NABL accreditation,^a^ and provides other requirements.
15 April 2020	Guidelines for COVID-19 testing in private laboratories
19 April 2020	Describes clusters of districts and which laboratory (including private labs) they are to be served by
25 April 2020	Fixed COVID-19 testing prices within private laboratories
27 April 2020	Released training module for laboratories on data management for COVID-19 (relevant to private labs)
2 June 2020	Revised links between districts and laboratories
12 June 2020	Set up sample collection centers for private laboratories
18 June 2020	Revised prices for Covid-19 testing prices within private laboratories
7 July 2020	Instructions regarding reporting of test results via unified COVID-19 portal
7 July 2020	Approval of TrueNat/CBNAAT COVID-19 testing^b^ including in private labs, so long as laboratories are NABL^a^ accredited
10 September 2020	Revised prices for COVID-19 testing prices within private laboratories

^a^ National Accreditation Board for Testing and Calibration Laboratories

^b^ COVID-19 testing platforms that use real-time RT PCR technology

The GOUP was slower to engage allopathic and AYUSH providers in the COVID-19 response. Private hospitals and providers were seen by the government as a *“back-up plan”* for treating COVID-19 (State level key informant 6). One district health administrator attributed the delayed engagement to the government’s inadequate capacity to monitor, manage, and control a vast private sector (District level key informant 2).

Early in the pandemic, when private providers were restricted from providing any COVID-19 healthcare services, district health authorities engaged with government-registered allopathic and AYUSH providers through remote platforms like WhatsApp groups. On these groups, district administrators shared general guidance about COVID-19 disease prevention, clinical management and facility disinfection practices, but also more targeted government orders and circulars about the reopening of the private facilities and the types of services they could provide. While allopathic providers appeared to receive relevant policy circulars and orders directly from the district health authorities or their local health association, some AYUSH providers described having no contact with district health authorities and instead relying on newspapers or their personal networks to stay informed.

Beginning in April 2020, district health authorities began convening trainings with allopathic and AYUSH providers, as well as dental surgeons, paramedical, and other private staff working in clinical settings. The trainings focused on preparing providers to safely reopen their health facilities, guiding them on how to organize triage areas for patients, record patient history, quarantine and test suspected cases, and use personal protective equipment (PPEs). AYUSH providers, who ran smaller practices, felt such trainings were more applicable to hospitals and nursing homes, not *"simple clinics"* like theirs (District-level private provider 12).

Besides trainings, in one study district allopathic providers also viewed biweekly district-level meetings as an avenue for information exchange between private providers and the district health authorities. Despite the meeting frequency, some allopathic providers described being confused about the latest policy guidelines, for example, the COVID-19 treatment guidelines. In comparison to allopathic providers, the level of engagement and participation of AYUSH providers during these district-level meetings appears to be minimal.

As the pandemic progressed in April and May, and government quarantine centers and health facilities became overwhelmed, the GOUP began to engage private nursing homes and hospitals to provide COVID-19 health services. Larger private sector establishments were prioritized by the government because they appeared to have the infrastructure and financial resources to meet government regulations. For example, in one study district, district health authorities repurposed a private teaching institute’s facilities to quarantine migrants. District health authorities also mandated private nursing homes and hospitals to allocate a portion of hospital beds for COVID-19 patients. In one district, allopathic providers met the government’s request by refurbishing an unused health facility to develop a new COVID-19 private hospital. Single doctor clinics of allopathic and AYUSH providers–which lacked the required infrastructure (e.g., availability of isolation wards or intensive care units) or the financial resources to meet government regulations–were not engaged in providing COVID-19 services.

Despite this, district health authorities appeared to involve all formal private providers in supporting the government’s contact tracing efforts. While all allopathic and AYUSH providers were expected to document and monitor their patients’ COVID-19 symptoms and contact information, the specific data requirements and reporting frequency varied by provider type. For example, nursing home and hospitals were required to provide daily email updates on a range of clinical (e.g., contact information of patients with suspected COVID-19 symptoms) and non-clinical indicators (e.g., number of staff working in the facility). In comparison, single-doctor clinics run by allopathic and AYUSH providers were required to document suspected COVID-19 patients to inform the district’s contact tracing efforts, but were not expected to submit daily reports to the district health authorities.

### Effects of government engagement on providers’ practices

When the lockdown was announced, only nursing homes and hospitals were allowed to provide emergency non-COVID health services, such as obstetric care. Though many respondents who ran nursing homes and hospitals chose to open, several facilities did so at a lower than normal capacity, due to limited access to PPEs and COVID-19 tests, as well as health worker fears of getting COVID-19. These facilities mainly provided emergency services to current patients, referring others who sought non-COVID-19 emergency services to government.

Financial reasons were also cited by a number of nursing homes that decided to remain closed during the lockdown. For example, a national-level order at the end of March 2020 prohibited any employers, including private hospitals and nursing homes from deducting or reducing salaries of their health staff to alleviate potential economic adversity [24]. Respondents described how the government closely monitored salary disbursements by requiring hospitals and nursing homes to submit financial statements, such as, bank account statements. Respondents indicated that smaller scale nursing homes that feared being unable to pay staff salaries, due to anticipated low patient load, chose to shut down, as one allopathic provider stated:

"*Since there were many problems related to the transportation of medicines*, *the rates of all the medicines were increased by 25%*. *We also had to give the salary to the staff for 3–4 months even when the clinic was closed because of the government order and out of humanity*." (District-level private provider 13)

Similarly, private laboratories interviewed were clearly aware of potential risks to their staff. They reported buying PPEs to equip their frontline staff responsible for sample collections, as well as paying them at increased rates for potential exposure to COVID-19, and providing their employees COVID-19 tests at half the cost.

Private nursing homes and hospitals that remained open during the lockdown described having to rely on their existing supplies of PPEs. In the initial month, many nursing homes self-organized and trained their own staff on how to use PPEs and safety protocols such as hand washing, social distancing, and how to disinfect and clean based on the guidelines that had been released by GOUP/GOI and ICMR. Guidelines issued by ICMR were initially seen as an important source of information since formal trainings with district health authorities began in April. Beginning in mid-April, at the direction of district health authorities nursing home and hospitals providing non-COVID-19 emergency services were required to modify their clinical practice based on government guidelines. A number of respondents established triage areas, isolation room, and flu counters based on the guidance they received from district health authorities, as one allopathic provider described:

*“First*, *we made sure that our biomedical waste segregation was very good*. *Second*, *we made sure that all the clinical staff*, *the reception and front desk staff knew how to use the thermal scanner*. *They had to wear the mask and place the protective shield*. *We made a proforma*, *a short questionnaire that was placed at the entrance gate of the hospital…*.*for anybody who gives the history of travel or history of fever or has fever*, *there was a triage area*, *so that the patients had to go first to the triage area and then to the isolation room…”* (District-level private provider 8)

Patients who presented at private facilities and were suspected of having COVID-19, were requested to get tested, however respondents explained that many patients were reluctant to get tested for fear of testing positive and having to quarantine in a public health facility. Nonetheless, nursing home and health facilities were required to submit daily reports with the contact information of suspected COVID-19 cases referred for testing. In addition, district health authorities requested daily reports on the number of staff working in the facility, and the daily frequency of cleaning, disinfecting the health facilities. Initially, private laboratories at the district level, shared results with the district’s Chief Medical Officer (CMO) via WhatsApp, but reporting of results became more organized over time, as the laboratories were instructed to report test outcomes via both the ICMR and the UP government portal.

Unlike nursing homes and hospitals, which were permitted to run at limited capacity, the government mandated that all outpatient services be suspended, implying that smaller clinics, which provide such services to shut down during the national lockdown until they were permitted to resume in June 2020. Initially, this meant that care for non-COVID-19 related outpatient services and all COVID-19 services could only be sought at government health facilities. However, several single–doctor AYUSH providers that had shut down their practices described continuing to see patients from their neighborhood as they felt that they could not turn them away. Such providers often relied on WhatsApp or phone calls to communicate with their patients. AYUSH providers, who described an increased demand in mental health services, made exceptions to see more serious patients face-to-face, as one AYUSH provider stated:

*“Often the patients have suicidal tendencies*, *and they feel they might die*, *so in that situation*, *we do their checkup*. *We sanitize everything and take them inside*, *use masks and check them*.*”* (District-level private provider 12)

In general, several other allopathic and AYUSH providers working in both hospital setting and private clinics settings described seeing their patients remotely. For example, one gynecologist/obstetrician described using WhatsApp to conduct antenatal care visits, sharing results of ultrasounds, providing prescriptions, and answering any patient concerns. To broaden their reach and to provide patients, particularly those with chronic conditions with medical advice, some allopathic and AYUSH providers also described publicizing their phone numbers through Facebook, Whatsapp and the local daily newspaper.

While our sample of RMPs is limited, it suggests a varied reaction to the initial lockdown. One RMP respondent, an RMP association representative, located close to Lucknow, indicated that he closed his office, as did other RMPs in the neighborhood during the initial lockdown. However, another RMP, an association representative and practitioner, noted that people continued to need health services, and while practices may have looked closed from the outside frequently RMPs were still providing services, particularly by phone and WhatsApp, and sometimes face-to-face. Both respondents concurred on the risks of providing services during the lockdown, noting in particular the dangers of police brutality, even for patients seeking care: “*they used to give the beatings first*, *and then they used to ask*" (State-level key informant 5).

After the lockdown, both RMP respondents offered services to patients with COVID-19, although this was expressly against government instructions. One respondent described “*providing vitamin C*, *cough syrups and other remedies to avoid "frozen mucus"”* (State-level key informant 5), whilst the other told COVID-19 patients to “*to drink warm water*, *take bath with warm water”* (State-level key informant 2). By and large such providers appear to have had very limited contact with the authorities, even for contact tracing. In one instance, one of the RMP respondents referred a patient whom they suspected of having COVID-19 to a government testing center, as they believed them to have had multiple contacts with other people, but this was not commonly the case.

Following the lockdown, in June 2020, all private facilities were permitted to provide outpatient services provided they meet required guidelines, for example, providers were trained in infection, prevention and control, and saw patients by appointment to minimize crowding. Despite being open, many single doctor clinics of allopathic and AYUSH providers noted a drastic decrease in demand for outpatient services compared to usual, in part due to the continuing travel restrictions. Several providers also felt that strict government policies as advertised through media created fear and reduced health seeking behavior among the population. Providers described serving the most severe cases, as one single doctor clinician explained: “*only those patients who have some serious problems which is sort of unavoidable*, *in that case they are taking an appointment and coming"* (District-level private provider 4). Similar low demand for health services were noted when nursing homes and hospitals were permitted to resume elective surgeries for non-COVID patients with a negative RT-PCR test in May/early June.

While private hospitals were requisitioned by the government to provide COVID-19 services or allocated a portion of their beds for COVID-19 patients beginning in April, it was only following the release of a government order on 10 July 2020 that private hospitals were allowed to directly admit, treat, and charge patients based on the outlined GOUP guidance [25]. One allopathic provider, who ran a large private hospital expected to see a surge of COVID-19 patients, particularly those enrolled in the Ayushman Bharat scheme, however this failed to materialize and the respondent attributed this to low COVID-19 positivity rates among this group.

### Barriers and facilitators to an effective private sector engagement

Experiences of public and private sector collaboration during the pandemic varied substantially by provider type. Unsurprisingly therefore, perspectives on the key barriers and facilitators to more effective private sector engagement also varied by provider type. Nonetheless, there were also some common themes that ran across providers. The themes distilled below–provider fear; communication; testing capacity; technology and case reporting; and regulation–address the issues raised by allopathic, AYUSH and diagnostic service providers. RMPs had such limited interaction with government that few barriers or facilitators were noted. As is often the case, providers tended to talk more about their frustrations (i.e. the barriers to collaboration) than the facilitators, but there were certain key aspects of the response–such as communication, testing capacity, and case reporting for example—where respondents described both positive facilitators as well as aspects that could be improved.

#### Fear

From the early days of the pandemic, fear was a major obstacle to more effective private sector engagement, as it likely was in the public sector too. Multiple respondents described how staff were initially unwilling to work and feared for their own health, as well as the implications for their family if they were to contract COVID-19 and die. One allopathic provider described how *“staff used to go on leave whenever they used to see any COVID-19 patient”* and recounted instances when hospitals guards would *“shoo the patients away and tell them to go to the medical college or district hospital*” (District-level provider 6). Strikingly these fears appeared to be fewer among more informal providers who often observed that while they took measures to protect themselves where possible (e.g., by using telemedicine), they felt bound to continue serving their clients.

The government provided PPEs and masks to private medical teaching hospitals and those facilities empaneled under Ayushman Bharat to provide COVID-19 services. Similarly, the government introduced the Pradhan Mantri Kalyan Yojana (life insurance) scheme for health workers in March 2020, however this was intended to cover only private sector health care providers who were drafted to participate in the response. To quell staff fears and encourage work, other private hospitals described providing financial incentives and increasing staff salaries, in addition to procuring PPE supplies themselves.

#### Communication

Most allopathic doctors appreciated efforts by local and state health authorities to disseminate policy guidelines, and provide trainings on infection prevention control protocols, as one allopathic provider stated:

*“It was good the information being given by the district authorities to us. They conducted the meetings and informed us about how to wear protective gear and protect yourself, then how to segregate the patient and when to refer, what are the basic modalities of the investigation to be done, when to treat…” (District-level private provider 10)*

However, many providers found the mode of communication (largely forwarding of WhatsApp messages, and ad hoc meetings and teleconferences in the district office) haphazard. Meetings were not always convenient for them, and there were no ready means to stay on top of policy guidance when there was no one-stop website or source of information that packaged all the current guidance. As one allopathic provider explained:

*“Making 100 or so WhatsApp groups and sharing information on the group is not sufficient*, *because WhatsApp group as*, *we both know that lot of people write so many other things*, *forwards*. *So the actual information gets lost*.*”* (District-level private provider 8)

In particular, allopathic providers described the confusion around COVID-19 treatment guidelines and inadequate communication at the district-level until the clinical management protocol for COVID-19 was streamlined in July 2020 [26]:

*“the information kept varying*, *the treatment kept varying*. *First we were using hydroxychloroquine*, *then ivermectin*. *Then suddenly ivermectin is now out*. *In one meeting the CMO had called the Medical College people and they said that you should use steroids*. *And in other meeting it was different”* (District-level private provider 8).

District health administrators confirmed that there was uneven communication of COVID-19 guidelines and recognized that there has been limited communication around guidelines specifically meant to benefit the private sector.

For AYUSH practitioners it appears that lines of communication were better established as the AYUSH Department had previously registered all AYUSH practitioners, thus facilitating communications:

*"All AYUSH practitioners are registered with a common board which was used to plan for training and deployment in case the situation demanded it and to for information sharing and communication*.*”* (State-level key informant 10)

Nonetheless AYUSH providers felt that direct communication with district health authorities was not strong and so they tended to rely more on communication within their own networks. The government appeared to have recognized this and also sought to engage with provider networks such as the UP branch of the National Integrated Medicine Association (NIMA) in order to stay connected with the private sector. Indeed, the role of provider associations, such as the Indian Medical Association, UP Nursing Home Association, as well as NIMA, appears critical in facilitating information flows between government and their members.

#### Testing capacity and COVID-19 hospital regulations

Early in the pandemic the government made a key decision to restrict care for COVID-19 patients to certain hospitals which were deemed to have adequate facilities. While in principle this policy helped ensure the quality of care provided, preventing hospital-acquired COVID-19 infections for example, in practice, shortages in testing capacity particularly during early days of the pandemic were a significant problem. While private health facilities were allowed to provide critical care and emergency services, they often did not have the testing capabilities to identify a COVID-19 versus a non-COVID-19 patient and consequently referred nearly all patients presenting to government hospitals, overwhelming the public health system. In one example, a private sector respondent conducted an emergency C-section before the result of the patient’s RT-PCR test was available. The patient received a positive test result after surgery, which forced the hospital to close down for two weeks leading both to negative financial impacts, as well as limiting care to other patients. Respondents from nursing homes and private hospitals in one district acknowledged receiving a one-time supply of free antigen testing kits from district health authorities in June/July 2020 which a provider described as “a big boon” allowing immediate testing and referral of suspected cases. However, patients were asked to pay for RT-PCR tests again when the supply ended (District-level private provider 8).

#### Technology and case reporting

Both AYUSH doctors and the private laboratories discussed barriers to reporting detected COVID-19 cases. AYUSH providers were aware that they were meant to report suspected COVID-19 cases to district authorities but in one of the study districts providers felt that there was no system to support that:

*"I do not think reporting was possible*. *We tried once or twice but we were not very well connected*. *I do not feel anybody must have called*. *And in case the patient felt he is really sick*, *he used to go by himself*.*"* (District-level private provider 12).

In the other study district however the reporting of suspected COVID-19 patients was made easy by the use of the technology, specifically the ’Amrit’ app which was established by the district magistrate (and later scaled up across UP). This made it simpler for private providers to upload patient details on the app which the district health team would then follow-up on.

Private diagnostic providers also referred to problems with reporting, but in their case technology had not eased the situation. While respondents accepted the need to report test results to government they indicated a high reporting burden, which was exacerbated by the need to report the same result across two different portals (one run by the ICMR and one by GOUP).

#### Government regulations and financial implications

Challenges with government imposed price regulation on COVID-19 related services were raised as an issue both by private allopathic providers and by the private laboratories. Private hospitals involved in delivering COVID-19 services noted that it was expensive to deliver such care, for example to incentivize staff to work with COVID-19 patients without offering bonuses. Safety measures also added to the costs, but often patient loads were low, and price caps also kept fees low.

Respondents from the laboratory sector also objected to the pricing regulations that were imposed by government, and in particular their frequent changes. The price cap on RT-PCR tests went from Rs 4,500 at the end of April, to Rs 2,500 in June to Rs 1,600 in September. Private providers complained that this eroded their profit margins, and indeed questioned whether it was feasible to make a profit at these rates. However, these gradual price reductions reflected decreasing manufacturing costs for COVID-19 tests and aimed to increase the accessibility and affordability of COVID-19 tests.

Besides price regulations, the government imposed other regulations intended to assure the quality of COVID-19 services provided in the private sector. For example, certain infrastructure requirements were put in place at any hospital treating COVID-19 patients, and these effectively meant that many smaller facilities or facilities that did not meet specific requirements were unable to provide such services. Private diagnostic firms noted the multiple layers of quality assurance and regulations, including the requirement to be accredited by National Accreditation Board for Testing and Calibration Laboratories, and to participate in the External Quality Assurance Services program. The government had a clear rationale for why such regulations should be imposed. For example, there were reports of private nursing homes in one of the study districts violating bio-waste management protocols, and apparently early on in the pandemic some of the private laboratories in UP were under scrutiny for false reporting. Specifically, two of the private laboratories issued negative test results for patients who later turned out to be positive (State-level key informant 1). While private sector providers typically accepted the need for regulations, some others described the heavy-handed tone from government that sometimes accompanied these requirements:

*“If the compliances are not met, then we can give you an NC [non-compliance notification] & we can shut you down. Such kind of threats were also there to the company." (District-level private provider 2)*

## Discussion

Our paper provides an empirical account of the private sector’s role in responding to the COVID-19 pandemic in UP, where the private health sector remains the primary source of care for the state’s roughly 240 million residents [12, 27]. During the COVID-19 pandemic, public health authorities in UP leveraged the capacities of different private health actors to bridge the gaps in essential and non-essential health services. Though initial government restrictions significantly limited the demand for and role of private health actors, the government rapidly engaged and mobilized private sector laboratories and diagnostic services, and simplified regulatory requirements to quickly onboard new private laboratories for COVID-19 testing. The government’s engagement of other private health sector providers, however varied considerably.

To provide surge capacity and deliver health services, the government engaged larger scale private hospitals and nursing homes, which were seen as better equipped to meet the government’s regulatory standards and infrastructure requirements as well as easier to manage and coordinate with compared to smaller-scale providers that were typically harder to identify, and more difficult to regulate. Consequently single provider AYUSH and allopathic private clinics largely acted as referral units and supported contact tracing efforts. In comparison, informal private sector providers, such as RMPs, who are more widespread and accessible to rural and poor communities [28], played no formal role in the COVID-19 response. Their illegal status restricted the government from engaging with them, with limited exceptions, where they supported district frontline public sector health workers in contact tracing efforts.

While the pandemic led the GOUP to identify, and document district-level private providers and their capacities, inadequate communication on the part of local authorities, poor strategies to engage private providers, and financial challenges among other things led to the under-utilization of this group in the COVID-19 response. These experiences mirror those of private providers in Ethiopia, who had been engaged to ramp up COVID-19 testing and to meet the surge in demand for COVID-19 services but struggled to make marginal profits [29]. Findings from other LMICs similarly show that private providers coped with these financial challenges by refusing services to COVID-19 patients, or filtering patients based on their capacity to pay, or evading government price ceilings until government authorities intervened [30]. The lack of engagement of smaller private health facilities was also observed during the 2014 Ebola outbreak. Private clinics in Guinea were less likely to be trained in infection control practices compared to public health facilities [31], and against the backdrop of an overwhelmed public health sector and unaffordable private establishments, poorer patients were forced to rely on informal providers for care [32].

In UP, there was also significant reliance on informal providers or RMPs, who are typically the first point of care in rural areas and for the poor [28, 33]. Although the GOUP did not acknowledge RMPs’ role in the COVID-19 response, a district in the neighboring state of Bihar offered COVID-19 trainings to informal providers [34]. However, this engagement was short-lived because it was quickly seen as “undermining the efforts of the State government” and led to the immediate suspension of a district officer” [35].

In general, many state governments in India have been reluctant to engage informal providers in the COVID-19 response because they are viewed to lack the appropriate knowledge required for treating patients. However, in West Bengal rural providers were officially recognized as health providers and trained to manage COVID-19 in rural settings [36]. A previous study in West Bengal also showed that training informal providers can improve case management [37], and other evidence suggests that the clinical performance of informal providers is comparable to providers with MBBS degrees [38, 39]. Ignoring RMPs may diminish rather than enable COVID-19 containment efforts. Sharing policy guidelines and engaging RMPs through trainings may better equip them to fulfill essential public health functions, such as, disseminating accurate health information with their patients, referring suspected cases to appropriate health facilities, and, addressing COVID-19 vaccine hesitancy. Furthermore, linking RMPs with other formal providers may help strengthen referral networks between rural communities and urban centers, where a number of COVID-19 health facilities exist. As WHO guidance on private sector engagement notes even informal providers “should be notifying cases, abiding with clinical protocols for testing, isolation and treatment, and ensuring financial and other barriers to care utilisation are eliminated” [40].

Our study also found that for smaller scale allopathic and AYUSH providers, professional medical and health associations served as critical intermediaries between the government and their vast membership. Associations disseminated government guidelines, conducted webinars, organized the bulk procurement of PPEs, and also provided specific COVID-19 treatment and management guidelines pertaining to different specialties [41]. While health association leaders also appeared to regularly communicate with the state government to receive information about the latest guidelines and to seek resolution on challenges their private providers experienced due to uneven implementation of guidelines by district health authorities, their district-level representatives felt left out of decision-making and consultative processes, even for the development of guidelines that would directly impact them, such as the financial caps for COVID-19 services provided in the private sector.

Strengthening the COVID-19 response and increasing pandemic preparedness at community levels would benefit from having stronger networks of small-scale private providers; establishing mechanisms to mobilize these networks and collaborations between public and private providers [42]; and ensuring two way communication of information so that private providers are not only aware of relevant health protocols and guidelines, but government also has a real time understanding of how its policies are playing out on the ground among this significant group.

In UP, some of these collaborations are already underway: public and private diagnostic networks, established as part of the COVID-19 response, may be leveraged to increase the reporting of notifiable diseases by the private sector in India, which has been a long-standing struggle [43, 44]. Similarly, district-level databases of private providers updated during the pandemic can be leveraged to bolster the government’s efforts to support COVID-19 vaccine roll-out, and mobilized to support pandemic preparedness at the community-level [45].

On a broader scale, strengthening the relationship between the public health sector and India’s vast, heterogenous private sector will be complicated. There has been a historically fractious relationship between the public and private health sector in India, which has been attributed to a complex set of barriers including differences in hierarchy, power, poor incentive structures, and conflicting values and goals [46, 47]. These challenges have limited the integration of the private health sector into the national response and have highlighted foundational issues that need to be addressed, such as what role should the private sector play in the national health emergencies; what capacity do they have, and what resources and inputs do they require to effectively partner with government; how much should they be reimbursed for health services, and how should the government protect them from financial losses [40].

In the field of HIV/AIDS there have been sustained efforts to involve private health care providers in the response, but also to provide them seats on advisory and governance committees thus helping to build trust and alignment [48]. Ideally these relationships between private and public sectors should not only be activated at times of crisis. Building relationships during periods of normality, including activities such as regularly collecting basic information about private health facilities, involving the private sector in key decisions relevant to them, as well as establishing agreements to provide surge capacity, can make government even better prepared during times of crisis.

There are a few limitations to the study. First, we had intended to include a larger group of small scale private providers from rural areas and informal providers in our sample, however due to the pandemic we were unable to travel to the study sites and sought to identify providers through existing databases and snowball sampling. Despite our best efforts, these approaches yielded relatively few interviews in rural settings or among informal providers which is unfortunate as these respondents may have provided deeper insights into government’s approach to engagement. Second, while the use of telephone or WhatsApp interviewing largely worked well, on occasion it meant that respondents were more uncomfortable than they perhaps would have been in a face-to-face setting. In these instances, it took longer for the research team to build rapport and encourage deep discussion. Finally, our data collection was conducted over the course of a few months against the backdrop of a very dynamic policy environment. Though we sought to reflect on emerging themes and identify new areas of inquiry as the research proceeded, this was sometimes challenging given the speed of change and we recognize that we may not have reached saturation on all points over time. Lastly, our study largely focused on Eastern UP districts, and additional interviews across other parts of UP may improve the transferability of these findings.

## Conclusions

The COVID-19 pandemic provided a unique opportunity to strengthen connections between the public and private health sector in India, one that may lead to long-lasting collaborations. The GOUP appeared to immediately engage and strengthen private laboratories and diagnostics services providers during the pandemic. Relatively slower to engage allopathic and AYUSH providers, the GOUP appeared to prioritize its engagement with larger hospitals and nursing homes, placing less emphasis on community-based providers, such as single doctor clinics. Strengthening the COVID-19 response at community levels, particularly in poorer and rural communities requires strengthening linkages with these provider networks, including RMPs, who remained an important source of care during the pandemic. A more systematic approach to integrating private providers into the health system is required, starting by understanding their capacities and then leveraging these effectively, both in times of normalcy and during emergencies, such as the pandemic. Strengthening routine communications between public and private sectors in a fashion that builds mutual trust and understanding is likely a prerequisite for such a systematic approach. We hope our findings provide a clearer view of the private sector’s experiences during the pandemic and can inform the development of effective approaches to engage the private sector at local and state levels.

## Supporting information

S1 Table Timeline of key policy measures highlighting the engagement between the Government of Uttar Pradesh and the private health sector during the COVID-19 pandemic in Uttar Pradesh, India. (DOCX)

S1 Text Inclusivity in global research. (DOCX)
