# Supplementary material for: Public sector engagement of private healthcare providers during the COVID-19 pandemic in Uttar Pradesh, India
Source: PLOS Glob Public Health. 2022 Jul 22;2(7):e0000750. doi: 10.1371/journal.pgph.0000750 (PMC10021290; doi:10.1371/journal.pgph.0000750)
Supplement: S1 Table — (DOCX) [file pgph.0000750.s001.docx]

**S1. Table 1.** Timeline of key policy measures highlighting the engagement between the Government of Uttar Pradesh and the private health sector during the COVID-19 pandemic.

1. Examples of key orders on building the facility-level infrastructure to support COVID-19 management across public and private health facilities

| **Date** | **Policy** | **Description** |
| --- | --- | --- |
| April 6, 2020 | Announcement of newly designated COVID-19 facilities | Two private hospitals designated as L1^a^, L2^b^ COVID-19 facilities - One L2 facility with 45 beds, 5 ventilators and one L1 facility with 35 beds, 0 ventilators. |
| April 9, 2020 | Announcement of newly designated COVID-19 facilities | Six private hospitals designated as L2 COVID-19 facilities with a combined capacity of 546 beds and 34 ventilators. |
| April 10, 2020 | Announcement of newly designated COVID-19 facilities | Six private hospitals designated as L2 COVID-19 facilities with a combined capacity of 368 beds and 33 ventilators. |
| May 5, 2020 | Notification of private hospitals as dedicated COVID-19 L-2 hospitals | Office order to notify 3 private sector hospitals in 3 districts as dedicated COVID-19 L2 treatment facility, each having a capacity of 100-150 beds and 1-3 ventilators. |
| May 20, 2020 | Notification of private hospitals as dedicated COVID-19 L-2 hospitals | Office order to notify 4 private sector hospitals in 4 districts as dedicated COVID-19 L2 treatment facility, each having a capacity of 60-120 beds and 1-3 ventilators. |
| June 21, 2020 | Notification of update of bed availability in all L1, L2, L3^c^ facilities | Notification to ensure that all COVID facilities (L1, L2 ,L3) update their bed availability and other functionality on the COVID19 portal. |

^a^L1 health facility catering to non-critical COVID-19 patients

^b^L2 health facility catering to more complicated COVID-19 cases

^c^L3 health facility catering to critical COVID-19 cases

1. Example of key orders focused on assessing current capacity and building additional capacity across the public and private health sectors

| **Date** | **Policy** | **Description** |
| --- | --- | --- |
| April 10, 2020 | Information on support from trained AYUSH^a^ doctors and paramedical staff | - The district authorities have been advised to take the support of the listed AYUSH master trainers (Medical officers and paramedics). - All the enlisted trainers from within the 75 districts and 19 AYUSH universities provided with online training. |
| April 14, 2020 | Directive to ensure compliance of instructions provided by the Government of India | - To address essential health services: state should undertake the mapping of all existing health facilities both private, not-for-profit, and public and designed them according covid-facilities and those that are meant to provide non-essential covid services. - Highlights the role of telehealth services, and opportunities for private providers to provide services where telehealth services are not accessible/available. - Hire/requisition non-government and private sector health workforce capacity, and more specifically by creating a web portal for empanelling human resources like private providers, retired professionals, etc. - Utilise AYUSH doctors in delivery of non-COVID essential services. |
| April 14, 2020 | Online training | - Online training through Zoom to private hospitals on Infection Prevention Protocol. |
| April 15, 2020 | Online training | - Online training through Zoom to private hospitals on Infection Prevention Protocol. |
| April 27, 2020 | Training on Infection Prevention and Control Protocol protocols | - Create Master trainers for Infection, Prevention and Control protocols, by training Nodal officers for all the district level medical units. Districts to form a district-level committee to ensure implementation of the Infection, Prevention and Control guidelines. - These Master trainers to provide cascading trainings on Infection, Prevention and Control protocols to all the hospitals - public and private - under them, such that all health workers at both public and private facilities in the district are trained. - The training will be conducted in phases; in the first phase Infection, Prevention and Control guideline training to nodal officers of at least 10 private hospitals from districts within the divisional head office and at least 5 private hospitals from other districts will be provided by April 29, 2020. - These private hospitals to begin providing emergency and essential services by April 30, 2020. - By May 5, 2020, trainings on Infection, Prevention and Control protocols must be ensured for all district level health facilities. |

^a^Ayurveda, Yoga & Naturopathy, Unani, Siddha, and Homeopathy providers, who are alternative medicine providers

1. Example of key orders focused on COVID-19 screening and testing strategies across the public and private health sectors

| **Date** | **Policy** | **Description** |
| --- | --- | --- |
| April 19, 2020 | Partnership with private labs for collection and testing samples | - Private labs (in addition to government) will be engaged to collect and test samples after ensuring quality assurance by ICMR^a^/VRDL^b^ network. Designated labs to provide daily updates to district, state, and central control rooms. |
| June 12, 2020 | Proper tracking of COVID-19 testing in the private sector | - Districts to ensure that all COVID-19 testing in the private sector is properly tracked, setting up dedicated sample collection centres and ensuring that all private labs update sample details and test results on the UP Covid19 tracks portal |
| July 7, 2020 | Quick approval of TrueNat/CBNAAT^c^ based COVID-19 test in private laboratories / clinics | - All private laboratories in the state who intend to initiate TrueNat/CBNAAT based testing for COVID-19 should be encouraged to immediately apply for NABL^d^ accreditation. |
| July 24, 2020 | RT-PCR^e^ test and static collection booth in private hospitals | - Guideline on setting up a mobile medical van to collect samples of all SARI^f^ patients in Private hospitals. Private hospitals are to be informed in advance to prepare a list of all SARI patients whose sample is to be collected by the mobile unit. |

^a^The Indian Council of Medical Research

^b^The Viral Research and Diagnostic Laboratories

^c^Cartridge Based Nucleic Acid Amplification Test

^d^National Accreditation Board for Testing and Calibration Laboratories

^e^Reverse Transcription Polymerase Chain Reaction

^f^Severe Acute Respiratory Infections

1. Example of key orders focused on health insurance coverage for healthcare workers involving in the COVID-19 response across the public and private health sector

| **Date** | **Policy** | **Description** |
| --- | --- | --- |
| March 28, 2020 | PM Gareeb Kalyan Package Insurance scheme to cover private sector too | - Private hospital staff, including retired, volunteers, local urban bodies, contract, daily wage, ad-hoc, outsourced staff, who are requisitioned to provide COVID-19 related responsibilities will also be covered. - Comprehensive personal accident cover of Rs. 50 lakh for 90 days and will also include accidental loss of life on account of contracting COVID-19. |
| June 19, 2020 | Reimbursement of private colleges as per the rules of Ayushman Bharat | - Expenses incurred for treating patients at designated COVID-19 private medical colleges and institutions to be covered under PM-JAY^a^ Ayushman Bharat. - Private colleges will be reimbursed as per the rules and payment slabs of Ayushman Bharat (i.e., Rs. 1,800 per day for General ward, Rs. 2,700 per day for high-density unit, Rs. 3,600 per day for Intensive Care Unit without ventilator, and Rs. 4,500 per day for Intensive Care Unit with ventilator). |

^a^National health insurance scheme also known as Ayushman Bharat Pradhan Mantri Jan Arogya Yojana or Ayushman Bharat National Health Protection Scheme. It was launched in September 2018 by the Government of India with an aim of offering healthcare coverage to 500 million citizens from low-income households. It provides an annual cover of Rs. 5 lakh (~US$7,000) per eligible family for medical treatment at any empanelled hospital, both public and private.

1. Example of key orders focused on ensuring the continuity of essential health services across the public and private health sector during the pandemic

| **Date** | **Policy** | **Description** |
| --- | --- | --- |
| April 11, 2020 | Statement ensuring continuity of essential health services, specially emergency and critical care services | - Hospitals and all related medical establishments to remain functional in private sector also, including dispensaries, chemist, pharmacies [including Jan Aushadhi Kendra)^a^, laboratories, pharmaceutical research labs, clinics, nursing homes, ambulance. |
| April 14, 2020 | Guidance note enabling delivery of essential health services during COVID-19 outbreak | - To address essential health services: state should undertake the mapping of all existing health facilities both private, not-for-profit, and public - Highlights the role of telehealth services, and opportunities for private providers to provide services where telehealth services are not accessible/available - Hire/requisition non-government and private sector health workforce capacity, and more specifically by creating a web portal for empanelling human resources like private providers, retired professionals, etc |
| April 19, 2020 | Guidance to private hospitals regarding arrangements before delivering non-COVID essential services | - Private Hospitals to ensure the following before making non-COVID essential services available: medical and Paramedic staff have been properly trained on COVID-19 and Infection Prevention and Control protocol; separate space for screening of patients is available and operational within the hospital; availability of Personal Protective Equipment and other safety material; disinfect/decontaminate of premised and inside of hospitals with 1% hypochlorite solution; ensure absolute adherence to management of biomedical waste |
| April 25, 2020 | Checklist of regulations and logistic arrangements to help District Magistrates of 18 worst affected districts with planning and preparation | - This checklist mentions that districts must provide a plan for the availability of dialysis, pregnancy, accidental cases, and cardiac emergencies services in the district through both the government and private sector. |
| April 27, 2020 | Initiation of emergency and essential services at private hospitals | - After receiving proper training on Infection Prevention and Control protocols, the selected private hospitals - at least 10 private hospitals from within the divisional head office and at least 5 private hospitals from other districts - can begin providing emergency and essential services by April 30, 2020. - By May 5, 2020 trainings must be provided at all district level health facilities. |
| May 13, 2020 | Directions for beginning emergency and essential services in private sector hospitals and nursing homes | - Notifies all Chief Medical Officers to ensure that emergency and essential services are started in all private sector hospitals and nursing homes. - Trainings of private sector staff on the Infection Prevention and Control protocols. - Additional guidance on ensuring the safe resumption of services - all private facilities empanelled under Ayushman Bharat (AB)^b^ as well as those facilities willing to provide services at AB rates will receive PPE kits and N-95 masks from the district |
| June 16, 2020 | Resuming outpatient department services in private sector hospitals and clinics in the state | - Regarding private clinics, only those with one/two physicians to start outpatient department services - Patients to be given prior appointments, as much as possible - To avoid unnecessary crowd at clinics, for everyone hour slot 4-5 patients to be informed beforehand |

^a^Special “kendras” or stores also known as Pradhan Mantri Bharatiya Janaushadhi Pariyojana Kendra or Jan Aushadhi Medical Store, that was first launched in 2008 (and later relaunched in 2015), in several districts across the country, to make quality generic medicines available at affordable prices for all.

^b^National health insurance scheme also known as Ayushman Bharat Pradhan Mantri Jan Arogya Yojana or Ayushman Bharat National Health Protection Scheme. It was launched in September 2018 by the Government of India with an aim of offering healthcare coverage to 500 million citizens from low-income households. It provides an annual cover of Rs. 5 lakh (~US$7,000) per eligible family for medical treatment at any empanelled hospital, both public and private.
